# Zoonotic diseases awareness and food safety practices among livestock farmers in Nepal

**DOI:** 10.3389/fvets.2024.1514953

**Published:** 2025-01-13

**Authors:** Deepak Subedi, Alok Dhakal, Sumit Jyoti, Sanjay Paudel, Ganesh Ranabhat, Ananda Tiwari, Ahmad I. Al-Mustapha

**Affiliations:** ^1^Department of Poultry Science, University of Georgia, Athens, GA, United States; ^2^Paklihawa Campus, Institute of Agriculture and Animal Science, Tribhuvan University, Bhairahawa, Nepal; ^3^Department of Health Management, Atlantic Veterinary College, University of Prince Edward Island, Charlottetown, PE, Canada; ^4^Faculty of Animal Science, Veterinary Science and Fisheries, Agriculture and Forestry University, Bharatpur, Nepal; ^5^Department of Food Hygiene and Environmental Health, Faculty of Veterinary Medicine, University of Helsinki, Helsinki, Finland; ^6^Department of Veterinary Public Health and Preventive Medicine, Faculty of Veterinary Medicine, University of Ibadan, Ibadan, Oyo, Nigeria; ^7^Department of Veterinary Services, Kwara State Ministry of Agriculture and Rural Development, Ilorin, Kwara, Nigeria

**Keywords:** food safety, livestock farmers, Nepal, preventive practice, risk, zoonosis

## Abstract

Interactions between humans and livestock could increase the risk of zoonotic disease transmission. In addition, limited knowledge of zoonoses and foodborne diseases among livestock farmers could heighten the risks of foodborne illness and outbreaks of zoonotic diseases. This study evaluated the awareness of zoonotic diseases and preventive practices for zoonotic and foodborne diseases among livestock farmers of the Chitwan, Rupandehi, and Tanahun districts of Nepal by conducting a cross-sectional survey of 280 livestock farmers. They were recruited using the purposive sampling method from October to December 2022. Descriptive statistics revealed that most (72.1%; *n* = 202/280) livestock farmers were aware of zoonosis. None of the farmers knew about the zoonotic nature of leptospirosis. Two-thirds of pig farmers (67%; *n* = 12/18) were aware of zoonotic transmission of swine flu, and more than half of the poultry (58%; 50/86) farmers knew about zoonotic avian influenza. The majority of the farmers who had dogs (83%) and cats (89.4%) in their homes or farms knew that rabies can be transmitted to humans from dogs or cats. The multivariable logistic regression analysis revealed that farmers from the Rupandehi district (aOR: 5.56; 95% CI: 2.18–14.22) and Chitwan (aOR: 6.52; 95% CI: 2.46–17.25) had a higher odds of having good preventive practices than those from Tanahun. Also, farmers who had no sickness in the past 6 months after consumption of animal products were three times (aOR: 2.98; 95% CI: 1.48–6.01) more likely to have better practices. Furthermore, secondary education (aOR: 3.64; 95% CI: 1.41–9.44) was a significant positive predictor of good zoonotic diseases and food safety preventive practices. Our study underscores the necessity to enhance Nepalese livestock farmers’ awareness and practices regarding zoonotic and foodborne diseases. It emphasizes the importance of understanding risks, effective behavioral change strategies, and engaging farmers in developing zoonotic disease and foodborne illness prevention programs.

## 1 Introduction

Zoonotic diseases, originating from animals and transmitted to humans, pose a significant global health threat. Approximately 60% of all infectious diseases are zoonotic, and up to 75% of newly emerging diseases have zoonotic origins (1). Recent pandemics, such as the COVID-19 and Mpox, are believed to have originated from animal reservoirs before human-to-human transmission (2–4). Human activities such as intensified agriculture and animal domestication have increased the risk of zoonotic diseases (5, 6). These zoonotic diseases whether endemic, epidemic, or pandemic, have far-reaching consequences for public health and the global economy (7).

Nepal is an agricultural country in South Asia where people interact closely with domestic animals and pets. Farming remains largely uncommercialized, with crop cultivation and livestock rearing being an essential activity for many Nepalese families. Many families grow crops and raise livestock for household needs, often relying on traditional farming methods, potentially increasing the risk of zoonotic disease (8). Livestock farming is increasingly recognized as a key driver for poverty reduction, ensuring food security and sustainable livelihoods (9). Farmers involved in livestock production are at elevated risk of zoonotic disease acquisition due to the varied nature and intensity of their animal interactions (10). The care and handling of animals, close contact with cattle or other animals, and consuming raw, uncooked, and tainted animal products are primary sources of human infections (11).

Additionally, farmers’ poor personal hygiene, lack of basic knowledge and practice regarding zoonosis, and disregard for biosecurity measures may contribute to the transmission of zoonotic and foodborne diseases (12). Similarly, farmers may be exposed to zoonotic infection from uterine discharge, an aborted fetus, and an infected placenta when they help the animals during parturition and neonatal care (13). Ethnicity, culture, and tradition can contribute to the vulnerability of specific farming populations to zoonotic diseases, as illustrated by practices such as consuming raw yak blood on special occasions in the Himalayan region and drinking cow urine in certain Hindu rituals. Other predisposing factors include the consumption of unpasteurized milk and undercooked meat is common in different ethnic groups of Nepal and increases the risk of foodborne illness caused by pathogens such as *Escherichia coli*, *Salmonella*, *Campylobacter*, *Staphylococcus*, and *Mycobacterium* (14–18).

In Nepal, where livestock holds significant economic, social, and cultural importance, frequent human-livestock interactions increase the risk of zoonotic infections (19). Diseases like avian influenza (20, 21), swine flu (22), anthrax (23), leptospirosis (24), zoonotic trematodes (25), helminths, and protozoal infections have been reported in both livestock and humans. However, the human disease burden and the ecology of these pathogens remain poorly understood, especially concerning their response to changing climates and land use (26). Additionally, although *Brucella* spp. and *Mycobacterium bovis* are common in livestock, their prevalence in humans is under-researched due to limited awareness and diagnostic capabilities (26). Six zoonotic diseases- Taeniasis/Cysticercosis/Neurocysticercosis, Leptospirosis, Hydatidosis, Brucellosis, Toxoplasmosis, and Avian Influenza– were designated as significant threats. In April 2021, the Nepalese Government updated the list to include Influenza (Avian and Seasonal), Rabies, Coronavirus (SARS-CoV and MERS-CoV, SARS-CoV2), Leptospirosis, Brucellosis, Salmonellosis, Leishmaniasis, Zoonotic Tuberculosis, Cestode (Cysticercosis/Hydatidosis) and Toxoplasmosis (27). Rabies has been reported to kill around 500 animals and up to 32 persons in recent years (28, 29) and recent rabies outbreaks in various domestic animals across western Nepal underscore the significance of our research (30).

Investigating the awareness of livestock farmers on zoonotic diseases can offer valuable insights into their knowledge of transmission routes and preventive measures. Evaluating the practices employed by livestock farmers concerning zoonotic diseases and food safety can identify gaps and shortcomings in current farming practices, hygiene measures, and disease surveillance systems. However, very few studies conducted in Nepal assessed awareness of zoonotic disease and food safety practices among livestock farmers (26, 31). Thus, this study was conducted to determine the zoonotic disease awareness and food safety preventive practices among livestock farmers in Nepal. Chitwan district has a high number of livestock farmers and is one of Nepal’s leading milk producers. Similarly Rupandehi district also have a high number of livestock farmers and border trade with India. Tanahun district was selected due to its geographical and economical difference with Chitwan and Rupandehi districts. This study can aid in customizing educational initiatives and interventions to promote safer farming practices, thereby mitigating the risk of zoonotic diseases, ensuring the health of both humans and animals and bolstering the sustainability of the livestock industry.

## 2 Methodology

### 2.1 Study design and study area

**Figure 1:**
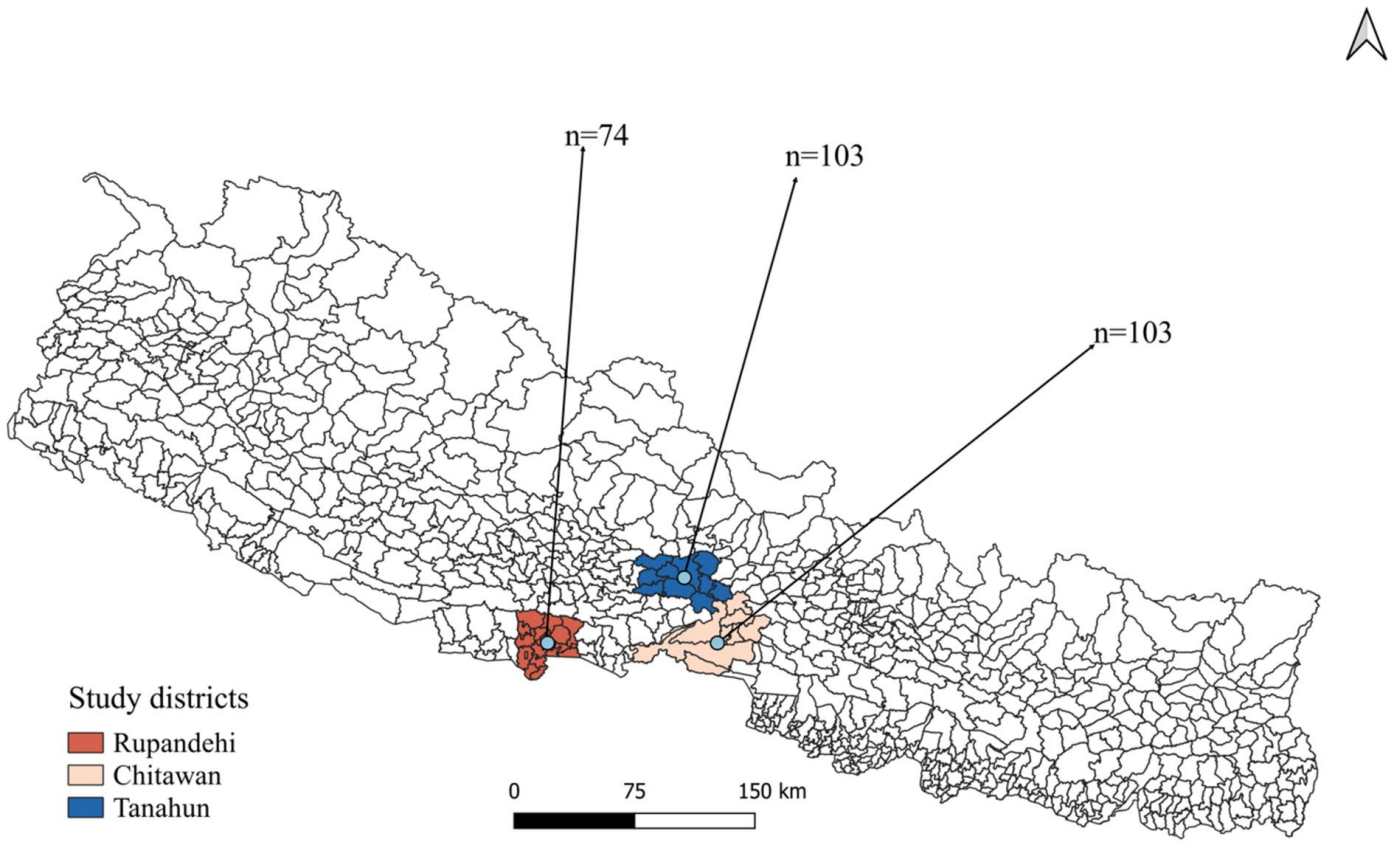
Map of Nepal showing districts from which the livestock farmers were recruited.

A cross-sectional study was designed to investigate livestock farmers’ awareness and practices toward zoonotic diseases and food safety in Rupandehi district from Lumbini province, Chitwan district from Bagmati province, and Tanahun district from Gandaki province of Nepal (Figure 1). From each district, more than 70 farmers were interviewed from October to December 2022. In this study, “farmer” refers to individuals engaged in farming or animal husbandry as either their primary or secondary occupation.

### 2.2 Study participants, sample size and sampling

Being a Nepalese citizen of age 18 years or above, residing within the country, and currently rearing livestock were used as the inclusion criteria for this study. Purposive sampling was used to enroll at least 70 farmers from each district. The primary data was collected using structured questionnaires to livestock farmers through face-to-face interviews. The objective and purpose of the study were initially described to the respondents during the interview, and the interviewer obtained their oral consent to participate in the study. The survey response was only obtained from those who provided consent and participated voluntarily in the study. To mitigate potential biases such as response bias, where participants may provide socially desirable answers, and recall bias, where participants may have difficulty accurately remembering past events, standardized questions, objective measures, short recall periods, cross-validation, and interviewer training were implemented.

### 2.3 Questionnaire design

The questionnaires were created based on a comprehensive literature assessment (26, 31) and the researcher’s knowledge. To improve its quality, the questionnaire was pre-tested among 20 farmers, but their responses were not included in the study. The questionnaire had sections for demographic information, types of animals reared, awareness of zoonotic diseases, and preventive practices for zoonotic diseases and food safety. The questionnaire was initially prepared in English and translated into Nepali for face-to-face interviews. The questionnaire was administered using the KoboToolbox and can be accessed here https://ee.kobotoolbox.org/x/OOYhnCfH.

### 2.4 Data analysis

#### 2.4.1 Data processing and descriptive analysis

The obtained responses from the online survey were imported into Stata software (version 15.1). The data were then examined for duplicate entries and inconsistencies. Frequencies and percentages were calculated for the demographics of the farmers and their awareness of zoonotic diseases. A graphical bar chart was used to display the status of livestock ownership within the study population. Additionally, the collected responses regarding farmers’ awareness levels of various zoonotic diseases and their disease prevention and control practices were presented in tabular format, showing the frequency of each response.

#### 2.4.2 Univariable and multivariable logistic regression

A univariable and multivariable logistic regression analysis was conducted to identify the association between demographic variables and practice levels. A scoring scale was devised since we needed a single practice-level variable representing the overall zoonotic disease and food safety practice standards. Eight practice questions (Table 3) were employed in developing this scale. A correct response was assigned a score of 1, while an incorrect response was given a score of 0. Each participant could achieve a score from 0 to 8 using this scale. Additionally, as followed by Subedi et al. (32), the threshold of 75% was established to convert the practice score into a binary scoring system, facilitating straightforward comparison and interpretation. Consequently, a score below six was categorized as “poor,” whereas a score of 6 or higher was classified as “good.” The independent demographic variables like farmer’s age, gender, education status, major occupation, farming experience, farmer illness status in the past 6 months, and herd size were considered for the univariable logistic regression model.

**Table 3:** Frequency table for the preventive practice of zoonotic diseases and food safety among Nepalese livestock farmers.

S.N.	Variable	Responses	Total	%
1.	Do you regularly vaccinate your animals against viral and bacterial diseases?	**Yes**	220	79%
		No	60	21%
2.	Do you cure your diseased animals with unorthodox ways of healing? (By using mantra or some spells)	Yes	130	46%
		**No**	150	54%
3.	Do you perform deworming on your animal?	**Yes**	225	80%
		No	55	20%
4.	How do you dispose of your dead animals?	**Burial**	246	88%
		Burning	34	12%
		Just drop off along the roadside or in water bodies such as rivers	12	4%
		Feed Other animals	25	9%
		Sell to butchers	8	3%
		Feed myself and my family with it	4	1%
5.	Do you drink raw or unboiled milk?	Yes	52	19%
		**No**	227	81%
6.	Do you eat undercooked or raw meat?	Yes	32	11%
		**No**	247	88%
7.	Do you wash your hands after having contact with animals?	**Yes**	243	87%
		No	37	13%
8.	Do you prefer walking barefoot at home, farm, or garden?	Yes	123	44%
		**No**	157	56%

In the table above, desirable responses for each practice question are highlighted in bold font and were used to develop the practice scale, as described in section 2.4.2.

##### 2.4.2.1 The equation for univariable logistic regression


(1) $$\mathrm{Logit}\;\left(p\right)=\beta_{0}+\beta_{1}x$$

In the Equation 1, the term “logit” refers to the natural logarithm of the odds of an event occurring. Here, *p* represents the probability of obtaining a good practice level, *β_0_* is the intercept, and *β_1_* is the coefficient of the independent demographic variable *x*. Independent variables (*x*) include demographic factors such as age, gender, education, district, experience, and occupation, as well as herd size and past illness history. Variables with a *p*-value <0.2 in the univariable logistic regression model were considered for inclusion in the multivariable logistic regression model.

##### 2.4.2.2 Equation for multivariable logistic regression


(2) $$\mathrm{Logit}\;\left(p\right)=\beta_{0}+\beta_{1}x_{1}+\beta_{2}x_{2}+\beta_{3}x_{3}+\cdots +\beta_{k}x_{k}$$

In Equation 2, *β*_0_ is the intercept, and *β*_1_ to *β*_k_ represents the coefficients of each variable (*x_1_* to *x_k_*) included in the model. Collinearity among the independent variables was assessed in the multivariable model using the variance inflation factor (VIF) utilizing the “collin” command in Stata. Interactions between independent variables were examined, and significant interactions were incorporated into the final model. The goodness of fit of the final multivariable model was evaluated using the Hosmer-Lemeshow test. The coefficients of the logistic models were exponentiated and presented as odds ratio (OR) in the result section. Both the univariable and multivariable model results are presented in the same table. Coefficients are presented as odds ratios (OR) for the univariable logistic model and as adjusted odds ratios (aOR) for variables that met the criteria for the multivariable logistic model.

## 3 Results

### 3.1 Demographic description of participants

**Figure 2:**
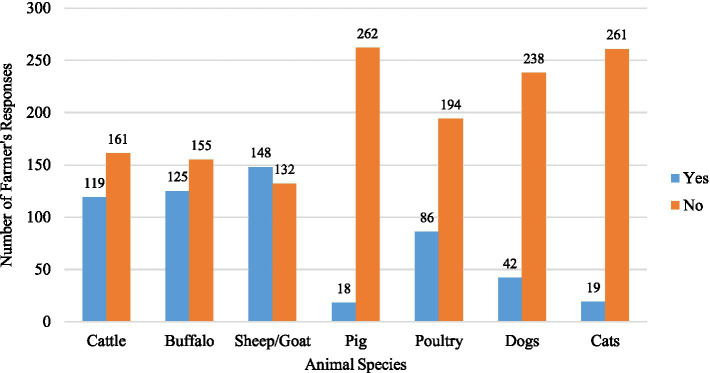
Bar chart showing the livestock ownership status (*n* = 280).

A total of 147 male and 133 female farmers participated in the study (Table 1). Among the participants, 38% were in the 50–59 age group, while 35% were aged between 30 and 39. Regarding the educational status of farmers, only 3.2% possessed tertiary education, while 38.9% had primary, and 32.9% lacked formal education. There were 103 farmers each from the Chitwan and Tanahun districts and 74 from the Rupandehi district. Livestock rearing was the primary occupation for the majority (53.6%) of the farmers, and about 10% had more than 30 years of farming experience. Family sizes varied, with around half (49.3%) of the farmers having five or fewer family members. One-fourth of the participants reported falling ill after recent contact with or consumption of animals or animal products. Approximately half (52.8%) of the farmers reported owning goats or sheep, while 42.5% owned cattle and 44.6% owned buffalo. A smaller percentage, 6.4%, mentioned owning pigs, and 30.7% had reared poultry. Additionally, 15% of the farmers reported having dogs, and 6.8% mentioned having cats on their farms or houses (Figure 2).

**Table 1:** Frequency table for demographic variables in awareness study of zoonotic diseases among livestock farmers of Nepal.

S.N	Variable	Category	Frequency (%)
1	Age	18–29	47 (16.8%)
		30–39	98 (35.0%)
		50–59	108 (38.6%)
		>60	27 (9.6%)
2	Gender	Male	147 (52.5%)
		Female	133 (47.5%)
3	Level of education	No formal education	92 (32.9%)
		Primary school	109 (38.9%)
		Secondary school	70 (25.0%)
		Tertiary education	9 (3.2%)
5	District	Rupandehi	74 (26.4%)
		Chitwan	103 (36.8%)
		Tanahun	103 (36.8%)
6	Primary occupation	Crop farming	26 (9.3%)
		Livestock rearing	150 (53.6%)
		Others	104 (37.1%)
8	Year of experience in farming	0–5	68 (24.4%)
		6–10	97 (34.8%)
		10–20	60 (21.1%)
		20–30	27 (9.7%)
		>30	28 (10.0%)
9	Number of members in the household	1–5	138 (49.3%)
		6–10	127 (45.4%)
		11–15	11 (3.9%)
		16–20	4 (1.4%)
10.	Have you ever fallen ill after contact or consumption of any animal or animal product?	Yes	70 (25.0%)
		No	210 (75.0)%

### 3.2 Awareness of zoonotic diseases

Out of 280 surveyed farmers, the majority (72.1%) knew that diseases could be transmitted from animals to humans (Table 2). However, among the 119 cattle and 125 buffalo farmers, only 31.9 and 30%, respectively, knew that cattle or buffalo can also be infected with rabies. None of the cattle and buffalo farmers knew the zoonotic nature of the leptospirosis. Similarly, only 31.1% of cattle and 27% of buffalo farmers knew bovine tuberculosis was a zoonotic disease. However, a significant proportion, 67% of pig farmers and 58% of poultry farmers were aware of the zoonotic nature of swine flu and bird flu, respectively. Only 16% of the poultry farmers were aware of the foodborne pathogens such as *Escherichia coli*, *Salmonella*, and *Campylobacter*. On a different note, most of farmers with dogs and cats were aware of the transmission of rabies to human from these animals.

**Table 2:** Frequency table for awareness of zoonotic diseases among livestock owners of Nepal.

S.N.	Variables	Responses	Total	%
1	Do you know that diseases can be transmitted from animals to humans?	Yes	202	72.1%
		No	78	27.9%
		Diseases	Yes	%
2.	Which of these diseases can you get from cattle? (*n* = 119)	Worms	48	40.3%
		Leptospirosis	0	0%
		Giardia/cryptosporidium	3	2.6%
		Bovine Tuberculosis	37	31.1%
		Anthrax	17	14%
		No diseases can be transmitted from cattle	1	<1%
		I do not know	18	15%
		Rabies	38	30%
3.	Which of these diseases can you get from buffalo? (*n* = 125)	Worms	45	36%
		Leptospirosis	0	0%
		Giardia/cryptosporidium	4	3%
		Bovine tuberculosis	34	27%
		Anthrax	19	15%
		I do not know	21	17%
		No diseases can be transmitted from buffalo	0	0%
		Swine flu	12	67%
4.	Which of these diseases can you get from pigs? (*n* = 18)	Ringworms including Taenia, Trichinella	5	28%
		Hydatidosis	3	17%
		I do not know	2	11.1%
		Bird Flu	50	58%
5.	Which of these diseases can you get from poultry (commercial or backyard)? (*n* = 86)	Foodborne pathogens such as *Escherichia coli*, *Salmonella*, *Campylobacter*	14	16%
		No diseases can be transmitted from poultry	2	2%
		I do not know	8	9%
		Worms	15	36%
6.	Which of these diseases can you get from a dog/cat? (*n* = 42)	Giardia/cryptosporidium	4	9%
		No diseases can be transmitted by dog	0	0%
		Rabies	17	40.5%
		Toxoplasmosis	2	4.8%
		I do not know	2	4.8%

### 3.3 Farmer’s disease prevention and containment practices

The majority of farmers in our study reported regular vaccination (79%) and deworming (80%) of their animals, respectively (Table 3). Almost half of the farmers (46%, *n* = 130/280) mentioned using unorthodox healing methods, such as mantras or spells, to treat their animals. While the majority of farmers mentioned burial and burning as methods for disposing of dead animals, a small percentage reported consuming dead animals (1%) and selling them to butchers (3%). Among the farmers surveyed, 11% reported consuming undercooked or raw meat, and 19% mentioned consuming raw or unpasteurized milk. While most farmers (87%) reported washing their hands after contact with animals, 44% preferred walking barefoot at home, on the farm, or in the garden.

### 3.4 Effect of sociodemographic factors on zoonotic diseases and food safety preventive practices among farmers

The results of univariable and multivariable logistic regression analysis are presented in Table 4. In the univariable logistic model, farmers’, education status, district, occupation, history of illness, and the herd size were significantly associated with good disease prevention and containment practices. However, in the multivariable logistic model, only the farmers’ district, and history of illness were significant, and education level was only marginally significant with the positive practice level (*p* = 0.045). Farmers from Rupandehi (OR: 5.56; 95% CI: 2.18–14.22) and Chitwan (aOR: 6.52; 95% CI: 2.46–17.25) had higher odds of having good preventive practices than farmers of Tanahun district. Farmers who were not sick within the last 6 months after contact with or consumption of any animal or animal product were three times more likely to have better practices of zoonotic disease and food safety than the farmers who were ill (aOR: 2.98; 95% CI: 1.48–6.01). Similarly, farmers with secondary education (aOR: 3.64; 95% CI: 1.41–9.44) were more likely to have good preventive practices than farmers with no formal education. Finally, farmers who were aware of the possibility of zoonotic disease transmission from their animals were more likely (OR: 4.2; 95% CI: 1.78, 9.92; *p* < 0.001) to have good practices of disease prevention.

**Table 4:** Univariable and multivariate logistic regression model of the preventive practice of zoonotic diseases and food safety among Nepalese livestock farmers.

			Univariable logistic regression		Multivariable logistic regression	
Characteristics	Practice level		OR (95% CI)	*p*-value	Adjusted OR (aOR) (95% CI)	*p*-value
	Poor (%)	Good (%)				
Age						
18–29	19 (40.43)	28 (59.57)	Ref	0.009	Ref	0.841
30–39	27 (27.55)	71 (72.45)	1.78 (0.86–3.71)		1.09 (0.45–2.67)	
50–59	20 (18.52)	88 (81.48)	2.99 (1.40–6.37)		1.42 (0.52–3.87)	
>60	12 (44.44)	15 (55.56)	0.85 (0.33–2.21)		0.95 (0.28–3.24)	
Gender						
Female	42 (31.58)	91 (68.42)	Ref	0.187	Ref	0.533
Male	36 (24.49)	111 (75.51)	1.42 (0.84–2.40)		1.23 (0.64–2.35)	
Education						
No formal education	36 (39.13)	56 (60.87)	Ref	0.014	Ref	0.045*
Primary school	29 (26.61)	80 (73.39)	1.77 (0.98–3.22)		2.01 (0.94–4.28)	
Secondary school	12 (17.14)	58 (82.86)	3.11 (1.47–6.57)		3.64 (1.41–9.44)	
Tertiary education	1 (11.11)	8 (88.89)	5.14 (0.62–42.87)		6.29 (0.56–70.43)	
District						
Tanahun	55 (53.40)	48 (46.60)	Ref	<0.001	Ref	<0.001*
Chitwan	12 (11.65)	91 (88.35)	8.69 (4.25–17.78)		6.52 (2.46–17.25)	
Rupandehi	11 (14.86)	63 (85.14)	6.56 (3.10–13.87)		5.56 (2.18–14.22)	
Experience (in years)						
0–5	18 (26.47)	50 (73.53)	Ref	0.97		
6–10	26 (26.8)	71 (73.20)	0.98 (0.49–1.98)			
11–20	17 (28.33)	43 (71.67)	0.91 (0.42–1.98)			
21–30	9 (33.33)	18 (66.67)	0.72 (0.27–1.89)			
>30	8 (28.57)	20 (71.43)	0.9 (0.34–2.40)			
Occupation						
Livestock farming	58 (38.67)	92 (61.33)	Ref	<0.000	Ref	0.289
Crop farming	4 (15.38)	22 (84.62)	3.47 (1.14–10.57)		2.84 (0.76–10.63)	
Others	16 (15.38)	88 (84.62)	3.47 (1.85–6.48)		0.99 (0.42–2.36)	
Herd size						
1–5	48 (34.78)	90 (65.22)	Ref	0.023	Ref	0.863
6–10	25 (19.69)	102 (80.31)	2.18 (1.24–3.81)		0.81 (0.20–3.27)	
11 and above	5 (33.33)	10 (66.67)	1.07 (0.35–3.30)		1.15 (0.56–2.37)	
History of illness (in the past 6 months)						
Yes	32 (45.07)	39 (54.93)	Ref	<0.001	Ref	0.0028*
No	46 (22.01)	163 (77.99)	2.91 (1.64–5.14)		2.98 (1.48–6.01)	

*p-values < 0.05.

## 4 Discussion

The agricultural sector in Nepal provides livelihoods for roughly 66% of the population, with the livestock industry representing a key economic component, accounting for approximately 11.5% of the total GDP and 25.7% of the agricultural GDP (33). Agricultural workers are more vulnerable to several zoonotic diseases due to their frequent direct contact with animals, increased exposure to environmental pathogens, and higher risk of encountering disease vectors in their work settings (34). Lack of knowledge about disease transmission from animals to humans and their prevention strategies can lead to higher exposures to multiple zoonotic diseases. Identifying the awareness status of zoonotic diseases and their prevention strategies can help design effective awareness and targeted intervention strategies, particularly in lower middle-income countries like Nepal. This study aimed to assess livestock farmers’ awareness and practices related to zoonotic diseases and food safety.

Our findings indicate that approximately 72% of livestock farmers knew zoonotic disease transmission from animals to humans. This figure is notably higher than those reported in previous studies in Nepal (45% by Kelly et al.) (26) and in Ethiopia (45.1%) (35). Education level was a significant predictor of preventive practice adoption, with tertiary and secondary educated farmers showing markedly better practices than those without formal education, a finding corroborated by earlier work on rabies knowledge in Nepal (36). Moreover, a regional effect was observed, with farmers in the more commercialized Terai region (Rupandehi and Chitwan) demonstrating a greater propensity for adopting preventative measures compared to farmers in the less developed hilly region (Tanahun). These geographical disparities are likely linked to variations in the commercialization and advancement of the agricultural and livestock sectors across the different districts. Interestingly, farmers who did not get sick after coming into contact with animals or animal products in the past 6 months were more likely to follow good practices than those who had been ill.

Public health data from the Department of Health Services in Nepal indicated that about half of the population are at high risk of rabies exposure, with an additional quarter at moderate risk (13). In our study, only 31.9% of cattle farmers and 30% of buffalo farmers were cognizant of the risk of rabies transmission from infected cattle or buffalo. However, a majority of farmers who owned dogs and cats demonstrated awareness of rabies transmission from these animals. The perception of dogs and cats as major sources of rabies is reinforced by the higher incidence of rabies outbreaks in these populations and the targeted focus of rabies control efforts (37, 38). Data from the Far Western region of Nepal, although not within the current study area, illustrates the breadth of rabies presence in the country; over a one-year period (27 November 2022 to 22 November 2023), rabies was confirmed in 41 dogs, 26 bovines (comprising 15 cattle and 11 buffalo), 12 goats, three jackals, and one cat (30). Consequently, while public awareness campaigns often target companion animals, it is imperative that farmers are educated on the susceptibility of livestock to rabies to ensure comprehensive risk mitigation strategies.

Furthermore, a significant proportion of pig farmers (67%) and poultry farmers (58%) demonstrated awareness of zoonotic nature of swine flu and bird flu, respectively. These findings align with Bagale et al. (31), who also identified avian influenza, rabies, and swine flu as the most recognized zoonotic diseases, while simultaneously noting a lack of knowledge regarding bovine tuberculosis, neurocysticercosis, and brucellosis. Notably, no cattle and buffalo farmers in our study were aware of the potential for leptospirosis transmission to humans. This finding is consistent with a study conducted in the Rupandehi district, also part of our research area, where none of farmers had knowledge of leptospirosis (24). Similarly, Kelly et al. (26) reported a significant lack of awareness concerning livestock-associated zoonoses such as leptospirosis, brucellosis, anthrax, and tuberculosis in Nepal. In our study, awareness of zoonotic nature of anthrax was limited to approximately 15% of the farmers. This proportion is higher than that reported in prior study in Nepal (4%), but lower than that found in Turkey (62.8%) (39). The level of awareness of common zoonotic diseases is likely to be influenced by several factors including lifestyle, disease burden, access to animal health care services, and the educational level of individuals.

Bovine tuberculosis (bTB), also known as zoonotic TB, primarily transmits to humans through the ingestion of raw meat, unpasteurized dairy products, and occupational exposure to cattle (40, 41). Nepal reported 70,000 cases of human tuberculosis in 2022, resulting in 18,000 fatalities (42). A study in Chitwan, Nepal, documented that 15% of animals in 60 households with human tuberculosis cases had bTB, and human patients were engaged in livestock-related activities such as feeding and milking (43). Despite bTB being endemic in Nepalese cattle populations, only 35% of cattle and buffalo farmers in our study were aware of its zoonotic potential. This finding contrasts with Kelly et al. (26), who reported a lower awareness (14%) among farmers across three districts of Nepal. In our study, 11 and 19% of farmers reported consuming undercooked or raw meat and raw or unboiled milk (unpasteurized), respectively, indicating a potential route of zoonotic transmission as well as increased risk of foodborne illness. Multiple studies have underscored the heightened risk of human tuberculosis associated with cattle exposure (44, 45). A study conducted in Nepal demonstrated that individuals with a history of exposure to sick cattle, consumption of raw dairy products, or cattle-rearing at homes had a fourfold increased likelihood of developing tuberculosis compared to those without these exposures (46). The necessity of a collaborative One Health approach, encompassing animal, human, and environmental health sectors, is paramount to mitigating the burden of tuberculosis in Nepal.

The 2019 recording of the first human death in Nepal due to avian influenza (H5N1) emphasized the existing zoonotic risk for poultry farmers and workers, as well as their heightened vulnerability (20, 21). In our study, approximately 60% of surveyed farmers were aware of zoonotic nature of bird flu, possibly attributable to its endemicity in Nepal (47). Similar awareness level was observed in previous study, where over 60% of respondents acknowledged a personal risk of avian influenza infection and expressed related concerns (48). These findings suggest that while awareness regarding avian influenza as a zoonotic disease is relatively widespread in Nepal, the frequent recurrence of outbreaks signifies a critical deficiency in the practical application of preventive measures among farmers and workers.

Our findings indicate a low level of awareness regarding toxoplasmosis as a zoonotic disease transmissible from cats, with only 10.5% of participants demonstrating knowledge of this disease. Given the potential for increased *Toxoplasma gondii* transmission through interaction with cats, particularly free-roaming, this lack of awareness is a significant public health concern (49). Notably, *T. gondii* is recognized as a major etiological agent in abortion among pregnant women (50).

In contrast to the low awareness of zoonotic risks, a substantial majority (80%) of farmers in our study reported routinely vaccinating their livestock. Unlike our result, Bagale et al. (31) reported that approximately two-thirds of respondents administered prophylactic vaccination to cattle. These discrepancies may reflect the diverse agro-ecological zones and varying access to veterinary services in different regions of Nepal. As highlighted by Kelly et al. (26), routine immunization against common diseases like foot and mouth disease is more prevalent in regions with intensive dairy production. In other areas, government-led mass vaccination campaigns are primarily reactive, initiated in response to outbreaks, rather than proactively implemented. Regarding other preventative measures, our survey revealed that 80% of farmers reported deworming their livestock. These results are consistent with observations from a study in the Bagmati province of Nepal, where the majority of farmers reported engaging in regular vaccination (63%) and deworming (59%) practices (51).

The majority of farmers in our study mentioned burial and burning as methods for disposing of dead animals, and a small percentage reported consuming dead animals (1%) and selling them to butchers (3%). Recently, a study conducted in Nepal by Bagale et al. (31) found that 12% of respondents disposed of dead animals in nearby rivers, a notably higher proportion than our finding of 4% who reported discarding carcasses on roads or in water bodies. These practices, including the consumption or sell of deceased animals and improper disposal in environment, poses a substantial threat to food safety and the potential spread of waterborne diseases.

The consumption of unpasteurized milk and raw meat is well-established as a risk factor for the transmission of zoonotic pathogens including *Brucella*, *Mycobacterium bovis*, *Salmonella*, and *Campylobacter*, as well as parasites such as tapeworms (44, 52–54). In our study, 11% of the farmers reported consuming undercooked or raw meat, and 19% mentioned consuming raw or unboiled milk. These findings necessitate targeted public health interventions, including campaigns to raise awareness of the risks associated with consuming unpasteurized milk and raw meat, to mitigate foodborne and zoonotic disease transmission.

Consistent with prior research in Nepal, approximately 90% of farmers in our study reported practicing handwashing after animal contact. This aligns with findings from the Chitwan, Tanahun, and Gorkha districts (26) and Manang, Tanahun, and Nawalpur districts (31). However 44% farmers in this study prefer walking barefoot at home, farm, or garden. These findings highlight a discrepancy between hand hygiene and footwear practices, underscoring the need for comprehensive hygiene education programs to mitigate the dual risks of farmer and public health hazards from contaminated products.

This cross-sectional study provides valuable insights into Nepali livestock farmers’ knowledge and practices regarding foodborne illness, though it captures only a single point in time. Future research should extend and replicate this study with a longitudinal approach to monitor changes over time and include qualitative methods to explore the social (access to veterinary care), economic, cultural (culture of eating raw meat and milk), and environmental factors (climate change, regular floods and landslides) influencing the adoption of zoonotic disease prevention practices. Given the limited awareness, particularly among illiterate and rural farmers, there is a pressing need for targeted training and outreach programs. Implementing these programs through a One Health approach could improve livestock health, enhance community well-being, and contribute to poverty reduction and national economic growth.

We acknowledge that this study has some limitations. Firstly, data collection relied on personal interviews, which may have led to socially desirable responses, potentially impacting response accuracy. Secondly, the use of purposive sampling could introduce bias, as participants may refer to others with similar characteristics or opinions. Finally, the participants of the study were limited to specific districts of Nepal, so the observed awareness and practices may not be representative of the entire country. Hence, the findings should be interpreted with caution.

## 5 Conclusion

Our study emphasizes the importance of enhancing the knowledge of Nepalese livestock farmers on zoonoses and food safety. It advocates for adopting both existing and new health practices to mitigate the risk of zoonotic pathogen transmission and improve food safety. Particularly, it is paramount to address the limited awareness observed among less educated and rural farmers. This highlights the need for on-site training and outreach initiatives tailored to diverse educational backgrounds and regions. Bridging the gap between awareness and practice will entail further investments in disease surveillance and collaborative efforts with farmers to develop tailored training programs focused on effective zoonotic and foodborne illness prevention and control measures.

## Data Availability

The original contributions presented in the study are included in the article/supplementary material, further inquiries can be directed to the corresponding authors.
